# Supplementary material for: Human vtRNA1-1 Levels Modulate Signaling Pathways and Regulate Apoptosis in Human Cancer Cells
Source: Biomolecules. 2020 Apr 16;10(4):614. doi: 10.3390/biom10040614 (PMC7226377; doi:10.3390/biom10040614)
Supplement: Supplementary file 1 [file biomolecules-10-00614-s001.zip › Bracher et al_Supplementary Data.pdf]

Supplementary Data

Human vtRNA1-1 levels modulate signaling pathways and regulate apoptosis in human cancer cells

Lisamaria Bracher[1,2], Iolanda Ferro[1], Carlos Pulido-Quetglas[2,3,4], Marc-David Ruepp[1,5], Rory Johnson[3,4] and Norbert Polacek[1,*]

[1] Department of Chemistry and Biochemistry, University of Bern, 3012 Bern, Switzerland
[2] Graduate School for Cellular and Biomedical Sciences, University of Bern, 3012 Bern, Switzerland
[3] Department of Medical Oncology, Inselspital, University Hospital and University of Bern, 3010 Bern, Switzerland
[4] Department of BioMedical Research, University of Bern, 3008 Bern, Switzerland
[5] United Kingdom Dementia Research Institute Centre at King's College London, Institute of Psychiatry, Psychology and Neuroscience, King's College London, Maurice Wohl Clinical Neuroscience Institute, London, UK

Supplementary Methods

Cell Culture

HEK293 and A549 cells were cultured identically to the HeLa and HEK293T cells (see main text).

vtRNA1-1 depletion

To reduce endogenous vtRNA1-1 levels in HEK293 and A549 cells, chemically modified chimeric antisense oligonucleotides (ASOs) were used (Supplementary Table 5). For that, cells were transiently transfected (JetPEI, Polyplus transfection) with 1 µg ASO (Exicon), directed against vtRNA1-1. As a control ASOs targeting vtRNA1-2 or a scrambled ASO were used. 24 hours post transfection, cells were collected by centrifugation (200 g, 5 minutes) stained with annexin V and analyzed with flow cytometry (see main text). Depletion was verified by northern blot (see main text). ASOs were designed as RNA/DNA/RNA chimeric oligonucleotides with a phosphorothioate backbone. Ten central deoxyribonucleotides are flanked by five 2'-O-methyl modified ribonucleotides on both sides.

Affinity purification of native in vivo assembled vtRNA-RNPs for mass spectrometry

RAT-tagged versions of vtRNA1-1 and vtRNA1-2 or the RAT-tag alone were stably transduced into the vtRNA negative Burkit Lymphoma cell line BL41 (lentiviral transduction, see main text). BL41 parental cells and the vtRNA or RAT-tag expressing derivatives thereof were cultured in RPMI 1640 growth medium, supplemented with 10% fetal bovine serum, 292 µg/ml L-glutamine and antibiotics (100 U/ml penicillin and 100 µg/ml streptomycin). For one affinity purification, 2 x 10^8 cells were resuspended in hypotonic lysis buffer (20 mM HEPES pH 7.4, 2 mM $MgCl_2$, 10% glycerol, 0.1% Igepal CA-630 and 1 mM DTT) and subjected to four cycles of snap freezing and thawing. Intact cell nuclei and mitochondria were removed by centrifugation (20'000 g, 20 minutes, 4°C). The microsomal

fraction including the vault complex was removed from the supernatant by another centrifugation step (100'000 g, 1 hour, 4°C) and this supernatant 100 (S100) was further separated on a 10% - 40% glycerol gradient (in RNP150 buffer, see below) during ultracentrifugation (28'000 rpm, 16 hours, 4°C). Total RNA was extracted (TriReagent protocol, Lucerna Chem AG), from the gradient fractions of a control sample and tested for vtRNA1-1 and vtRNA1-2 expression by northern blot (see main text). In accordance with the control gradient, vtRNA1-1/vtRNA1-2 positive fractions were pooled, the glycerol removed, and the extracts concentrated using Amicon Ultra-4 centrifugal filter devices (3 kDa MWCO) by centrifugation (7'500 g, 2 hours, 4°C). 1 mg protein of the concentrated RNP extract was incubated with 1.5 µg/ml *Pseudomonas aeruginosa* phage 7 coat protein (PP7CP, kindly provided by Thomas Hörnes; A. Hüttenhofer lab at the Medical University Innsbruck, Austria) in a total volume of 1 ml RNP150 buffer (20 mM HEPES pH 7.4, 2 mM $MgCl_{2,}$ 150 mM NaCl, 1% Igepal CA-630 and 1 mM DTT) for 2 hours at 4°C under constant rotation. As a control for unspecific binding to the beads, in one sample no PP7CP was added that was otherwise treated equally. 50 µl washed (RNP150 buffer) and blocked (Li-Cor blocking buffer, 0.1 µg/µl yeast bulk tRNA) magnetic rabbit mAb IgG Isotype control beads (Cell Signaling Technology) were added to the pre-incubated RNP-PP7CP extracts. Affinity purification was performed for 2 hours, 4°C and constant rotation. Subsequently, beads were washed twice in RNP150 buffer and twice in RNP wash buffer (50 mM Tris-HCl pH 8.0, 50 mM NaCl and 0.1% Igepal CA-630). Beads were immediately submitted to the Proteomics and Mass Spectrometry Core Facility of the University of Bern where the proteins were digested from the beads and further processed for mass spectrometry analysis according to their internal standard pipeline. LC-MS/MS was performed using a nano-UPLC and an orbitrap mass spectrometer. Proteins pulled-down with the RAT-tag alone or with the IgG beads were removed from the two experimental datasets. The top 200 identified proteins pulled-down with RAT-tagged vtRNA1-1 or vtRNA1-2 were compared and overlapping proteins removed from the vtRNA1-1 specific dataset, resulting in 73 possible vtRNA1-1 binding proteins that were subsequently subjected for gene ontology (GO) analysis to the online database for annotation, visualization and integrated discovery (DAVID).

Supplementary Tables

Supplementary Table S1. Single guide RNA seed sequences targeting the vtRNA1-1 and the vtRNA1-3 loci.

sgRNA ID	Score	Seed sequence 5' - 3'	PAMseq	Direction
Hs_HVG1 up1	77	G TAAACCGGGGACACACCTG	CGG	→
Hs_HVG1 up2	76	G AGAAGGAGACAGTAAACCG	GGG	→
Hs_HVG1 dw1	68	G AACCGCCCAGAGAGGTAAA	AGG	←
Hs_HVG1 dw2	79	G CGAGAAGATACAAGTATAC	AGG	←
Hs_HVG3 up1	86	G CCTGGAGATAGCGGCGGAT	TGG	→
Hs_HVG3 up2	78	G CAATTGCAGAGTGTCCATC	TGG	←
Hs_HVG3 dw1	82	G GCTGATTTTAGTAGTGGGC	AGG	→
Hs_HVG3 dw2	61	G AAATGGCTGATTTTAGTAG	TGG	→

Supplementary Table S2. Oligonucleotides for the cloning of vtRNA1-1 and vtRNA1-3 locus targeting pCRISPR-EF1α-eSpCas9(1.1) and pMB1610-pRR-puro constructs.

Oligo ID	Sequence 5' - 3'
U6-EcoRI fwd	ggtggtG/AATTCGAGGGCCTATTTCCCATGATTCC
gRNA-XbaI rev	ggtggtT/CTAGAAAGCACCGACTCGGTGCCACTTT
HVG1-us2_SalI-fwd	aacaacG/TCGACGAGAAGGAGACAGTAAACCG
HVG11-ds2_SpeI-rev	aacaacA/CTAGTGCGAGAAGATACAAGTATAC
HVG1up1-SalI fwd	aacaacG/TCGACGTAAACCGGGGACACACCTGCGG
HVG1dw1-SpeI rev	aacaacA/CTAGTGAACCGCCCAGAGAGGTAAAAGG
HVG3up2-SalI fwd	aacaacG/TCGACCCAGATGGACACTCTGCAATTGC
HVG3dw1-SpeI rev	aacaacA/CTAGTCCTGCCCACTACTAAAATCAGCC

nnnnnn = extra nucleotides and N/NNNNN = restriction sites for *EcoRI*/*XbaI* or *SalI*/*SpeI*

Supplementary Table S3. Primer used for the generation of vtRNA1-1 mutants.

Mutant	Oligonucleotide	Sequence 5' - 3'	Cloning Strategy
M1	Hs_HVG1centBulgemut-QC-f	CGAACAACCCAGACAGGTTGCGGTGGCCAATTAAAGAACTGTCGAAGTAACCGCTGAGCT	QuickChange mutagenesis
	Hs_HVG1centBulgemut-QC-r	AGCTCAGCGGTTACTTCGACAGTTCTTTAATTGGCCACCGCAACCTGTCTGGGTTGTTCG	
M2	Hs_SL2trunc-rev	P-TTGTGCTTGTTTCAATTAAAGA	PCR
	Hs_SL2trunc-fwd	P-AAGTGTTCGAGACCCGC	
M3	mPIPE-SL2stemdel-fwd	CAAGCA×CGTCTG×TGTTCGAGACCCGCGGGCGCTCTCCAG	PIPE cloning
	mPIPE-SL2stemdel-rev	CGAACA×CAGACG×TGCTTGTTTCAATTAAAGAACTGTCG	
M4	mPIPE-SL2loopmut-fwd	GCAACCCCAGAGGGTTGTTCGAGACCCGCGGGCGCTCTC	PIPE cloning
	mPIPE-SL2loopmut-rev	ACAACCCTCTGGGGTTGCTTGTTTCAATTAAAGAACTGTC	
M5	mPIPE_53_55-fwd	AACAAGCATCGTGTCTGGGTTGTTCGAGACCCGCGGGCGCT	PIPE cloning
	mPIPE_53_55-rev	CCAGACACGATGCTTGTTTCAATTAAAGAACTGTCGAAGTAAC	
M6	mPIPE_62_64-fwd	CGTGTCTGCGATGTTCGAGACCCGCGGGCGCTCTCCAGTCC	PIPE cloning
	mPIPE_62_64-rev	TCGAACATCGCAGACACGATGCTTGTTTCAATTAAAGAACT	
M7	Hs_mPIPE_32_45-fwd	CACATCTTTAATTCATACAAGCAACCTGTCTGGGTTGT	PIPE cloning
	Hs_mPIPE_32_45-rev	GTATGAATTAAAGATGTGTCGAAGTAACCGCTGAGCTA	
M8	Hs_mPIPE_30-40-fwd	TCGAGTCAAGAAATTTTGAAACAAGCAACCTGTCTGGG	PIPE cloning
	Hs_mPIPE_30-40-rev	CAAAATTTCTTGACTCGAAGTAACCGCTGAGCTAAAGC	

P- = 5' phosphate; NNN or NNN = mutated nucleotides; × = nucleotide deletion; NNNNNNNN = 18 overlapping nucleotides between primer pairs (required for PIPE cloning)

Supplementary Table S4. Northern blot probes.

Probe ID	Sequence 5' - 3'
vtRNA1-1_NB	GCTTGTTTCAATTAAAGAACTGTCG
vtRNA1-3_NB	GAGGTGGTTTGATGACACGCGAA
5.8S rRNA_NB	TCCTGCAATTCACATTAATTCTCGAGCTAGC
Hs_vtRNA1-1_M1_NB	CAGACAGGTTGCTTGTTTTCGAAGT
Hs_vtRNA1-1_M2_NB	GCGGTGGCCAATTAAAGAACTGTCG
Hs_vtRNA1-1_M8_NB	CTTGTATGAATTAAAGATGTGTCG
Hs_vtRNA1-1_M9_NB	CTTGTTTCAAAATTTCTTGACTCG
U1 snRNA probe	CAATGGATAAGCCTCGCCCT
U2 snRNA probe	TTCCATCTCCCTGCTCCAAA
U4 snRNA probe	CGCCTCGGATAGACCTCATT
U6 snRNA probe	ATTTGCGTGTCATCCTTGCG

Supplementary Table S5. Chemically modified ASO for vtRNA depletion

Oligo ID	Sequence 5' - 3'
vtRNA1-RH2-3_modif	mU*mG*mU*mU*mU*C*A*A*T*T*A*A*A*G*A*mA*mC*mU*mG*mU
ASO_vt1-2	mA*mG*mA*mG*mG*T*G*G*T*T*A*C*A*A*T*mG*mU*mA*mC*mU
ASO_Scrambled	mG*mU*mA*mU*mU*T*A*C*A*A*T*T*G*A*C*mG*mU*mA*mU*mA

m = 2' - O - methyl modified ribonucleotides; * = phosphorothioate backbone

Supplementary Figures

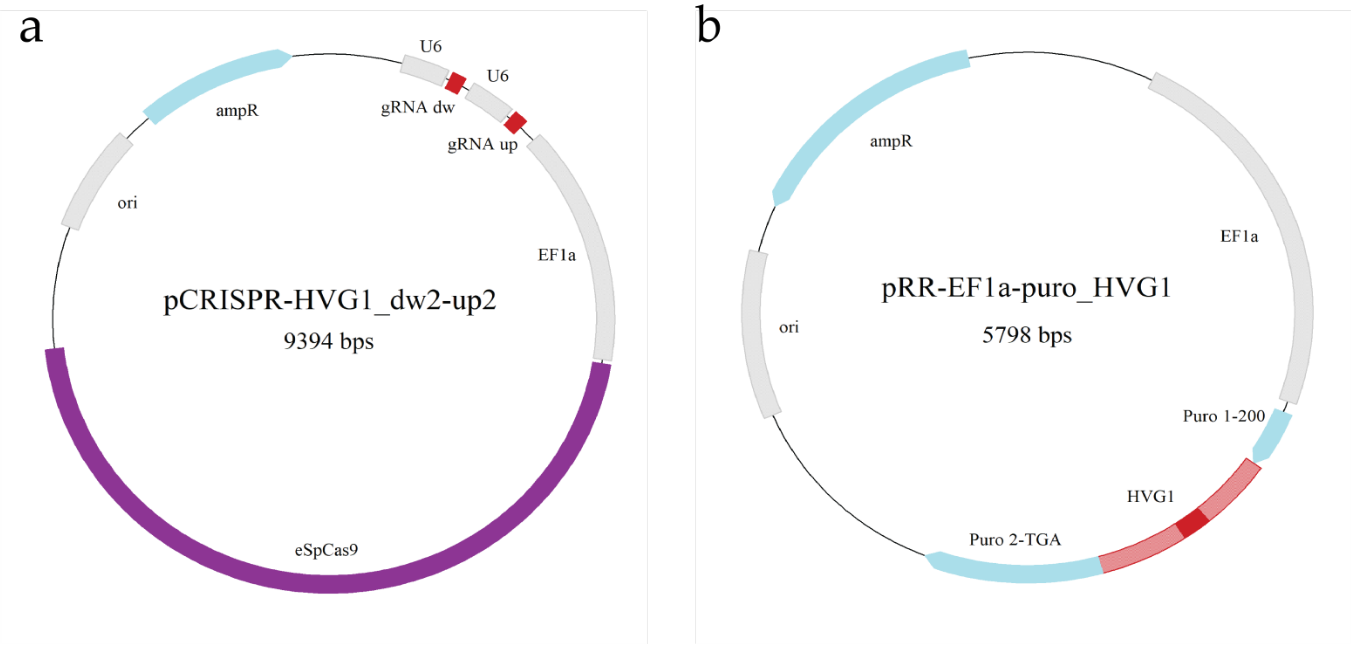

Supplementary Figure S1: Plasmid maps of constructs used for CRISPR-based genome editing. (a) Plasmid map of a representative pCRISPR construct targeting the vtRNA1-1 gene. The plasmid encodes two guide RNAs (gRNA) under the control of individual U6 promoters (U6). One gRNA targets the upstream genomic region of the vtRNA1-1 gene (gRNA up) and the second gRNA targets the downstream genomic region of the vtRNA1-1 gene (gRNA dw). An enhanced specificity Cas9 (eSpCas9) gene is under the control of the EF1α promoter. **(b)** Plasmid map of a representative pMB1610-pRR-EF1α-puro selection construct for genomic cleavage in the region of the vtRNA1-1 gene. The genomic sequence between the two targeted PAM sites up- and downstream of the vtRNA1-1 gene were inserted between an interrupted puromycin resistance open reading frame (ORF) that is controlled by an EF1α promoter. Genomic Cas9 cleavage will also cleave this insert, restore the puromycin ORF and mediate temporary puromycin resistance to transfected cells. Grey: promoter or origin of replication, magenta: eSpCas9, cyan: ampicillin or puromycin resistance, red: gRNA or genomic insert.

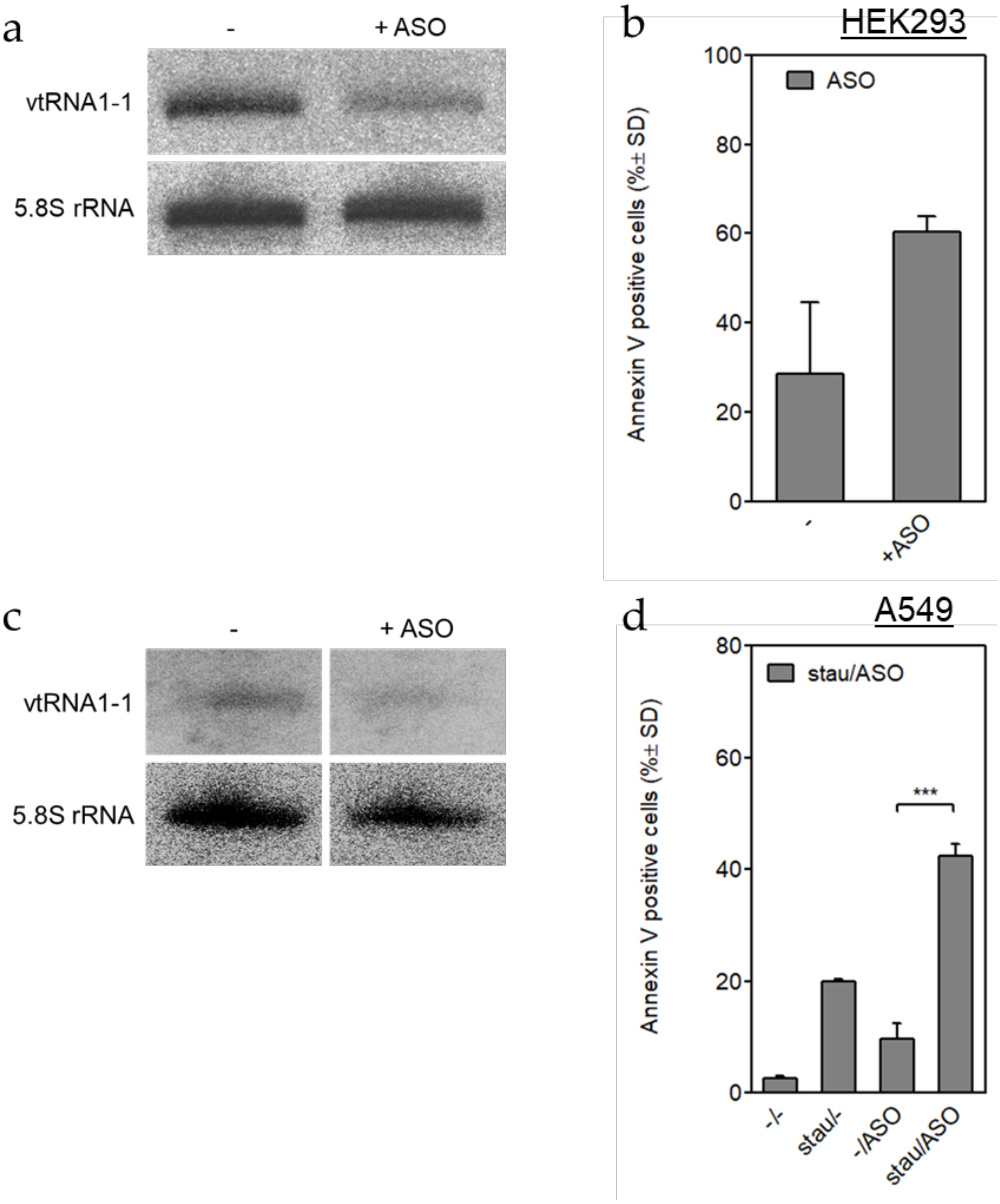

Supplementary Figure S2. vtRNA1-1 depletion results in more spontaneous cell death in HEK293 and A549 cells and is further increased in staurosporine challenged A549 cells. (a) Northern blot analysis reveals the amount of ASO-mediated vtRNA1-1 depletion in HEK293 cells 24 hours post transfection. 5.8S rRNA served as loading control ((-) no ASO control). **(b)** FACS quantification of annexin V stained cells of the same vtRNA1-1 depleted HEK293 cells ((-) no ASO control). **(c)** Northern blot analysis of the ASO-mediated vtRNA1-1 depletion in A549 cells. 5.8S rRNA served as loading control ((-) no ASO control). **(d)** FACS analysis of the same A549 cells treated without vtRNA1-1 depletion but with 24 hours staurosporine treatment (-/-, stau/-) or treated with a combination of vtRNA1-1 knock-down and 24 hours stauropsorine (-/ASO, stau/ASO). Significant differences were determined using the two-tailed unpaired Student's t-test (***P<0.0001).

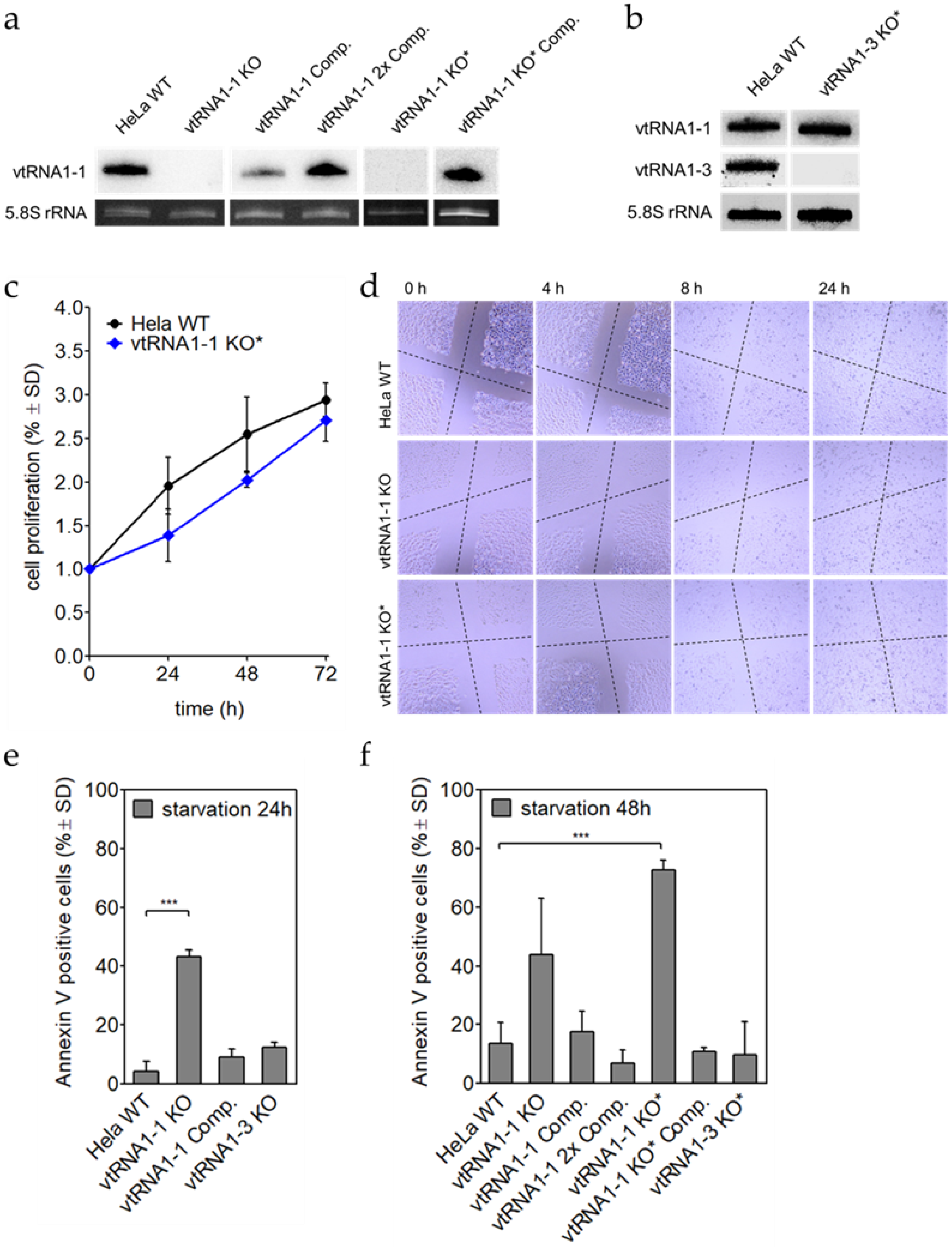

Supplementary Figure S3. Different CRISPR/Cas9 single cell clones of vtRNA1-1 knock-out and vtRNA1-3 knock-out display the same apoptosis phenotype. (a) Northern blot analysis of vtRNA1-1 complementation cells (vtRNA1-1 Comp., vtRNA1-1 2x Comp.) and a second vtRNA1-1 knock-out clone and its corresponding complementation cell line (vtRNA1-1 KO*, vtRNA1-1KO* Comp.). 5.8S rRNA served as loading control. **(b)** Northern blot analysis of a second vtRNA1-3 knock-out clone (vtRNA1-3 KO*). 5.8S rRNA served as loading control. **(c)** MTT assay comparing HeLa WT and vtRNA1-1KO* proliferation rates for 72 hours. **(d)** Migration assay over a time period of 24 hours of HeLa WT

and the two vtRNA1-1 knock-out clones vtRNA1-1 KO and vtRNA1-1 KO*. **(e)** FACS quantification of annexin V stained cells following 24 hours of starvation with chloroquine. **(f)** FACS quantification of annexin V positive cells including the three different vtRNA1-1 complementation cell lines (vtRNA1-1 Comp., vtRNA1-1 2x Comp., vtRNA1-1 KO* Comp.), the two vtRNA1-1 knock-outs (vtRNA1-1 KO, vtRNA1-1KO*) and an additional vtRNA1-3 knock-pout (vtRNA1-3 KO*) after 48 hours of starvation with chloroquine. Significant differences were determined using the two-tailed unpaired Student's t-test ***P<0.0001.

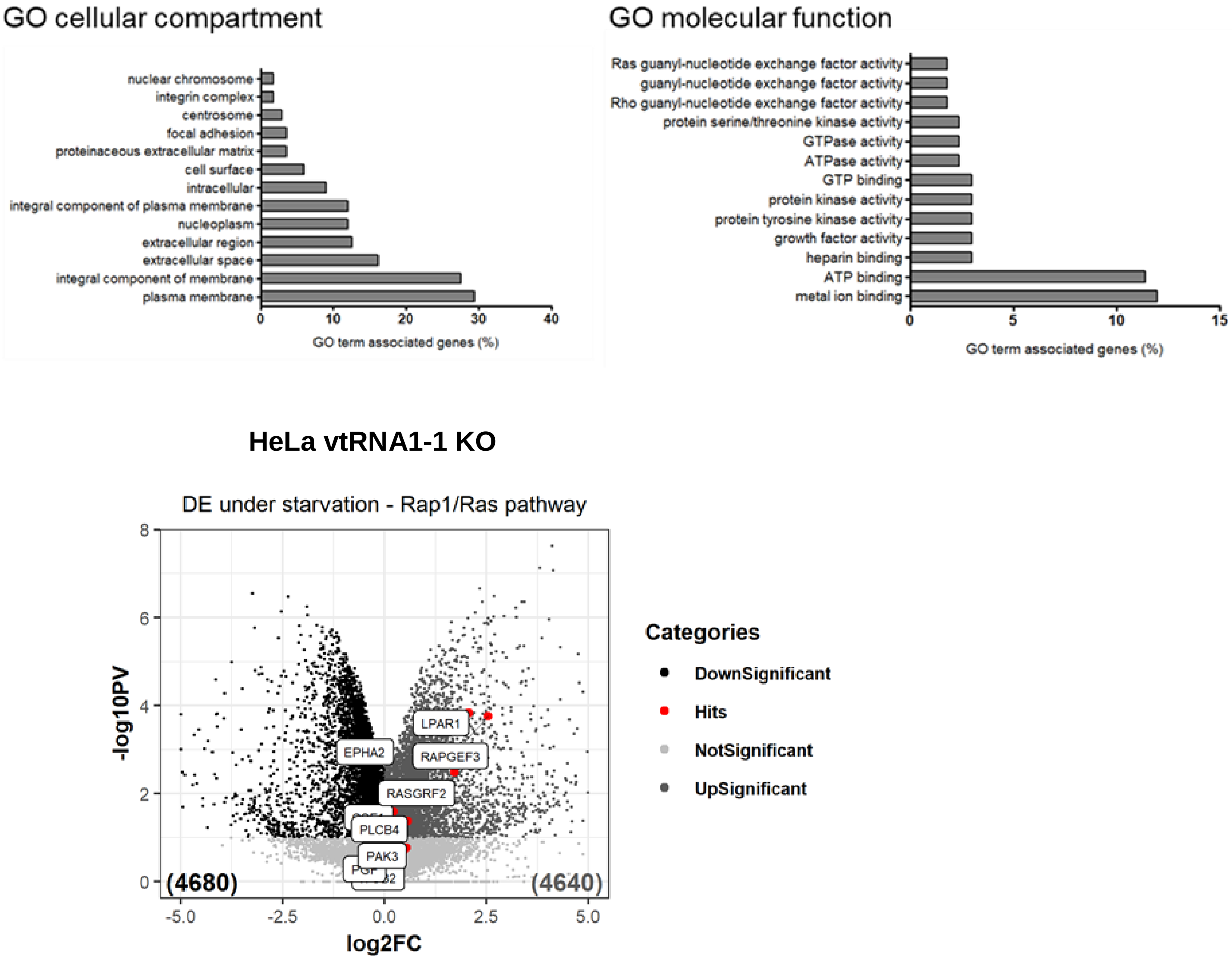

Supplementary Figure S4. Gene ontology analysis of the differentially expressed genes in the vtRNA1-1 knock-out cell line under starvation. Database for annotation, visualization and integrated discovery (DAVID) gene ontology (GO) analysis performed on 167 genes differentially expressed specifically in the vtRNA1-1 knock-out cells following 24 hours of starvation with chloroquine. The functional annotation clustering application was used to determine important GO terms. The volcano plot (bottom) of DE genes in HeLa vtRNA1-1 knock-out cells under starvation illustrates the genes annotated to the Rap1 or the Ras signaling pathways. The genes LPAR1, RAPGEF3 and RASGRF2 are significantly upregulated.

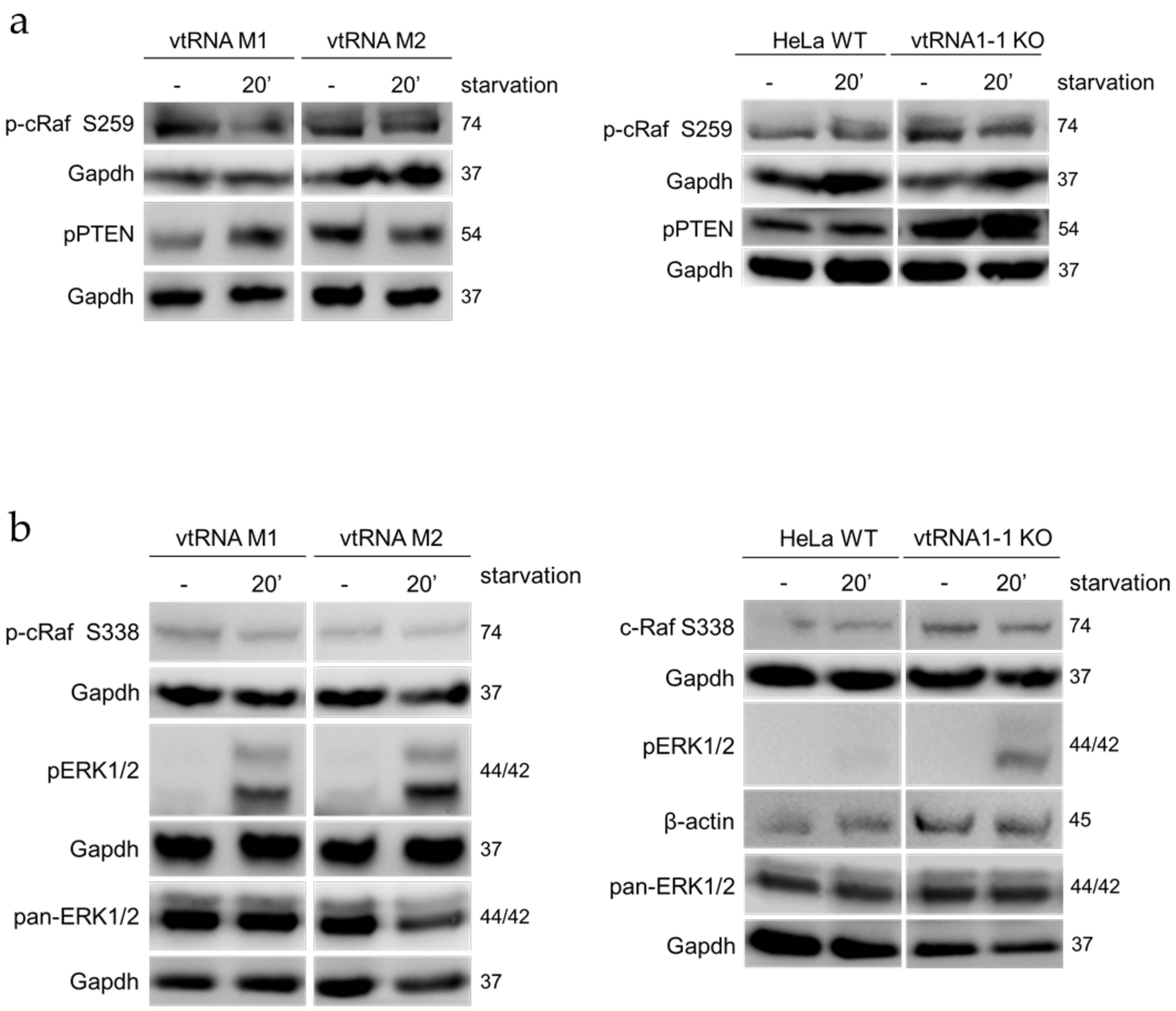

Supplementary Figure S5. vtRNA1-1 mutations M1 and M2 demonstrate a similar PI3K/Akt and ERK1/2 pathway misregulation like the vtRNA1-1 knock-out cells (a) Western blot analysis was used to assay the levels of PI3K/Akt involved Ser259 phosphorylated c-Raf and Ser380 phosphorylated PTEN as a result of 20 minutes serum starvation with chloroquine. Gapdh served as loading control. **(b)** Western blot analysis revealed the phosphorylation state of the ERK1/2 pathway involved c-Raf at Ser338 and ERK1/2 at Thr202/Tyr203, following 20 minutes of serum starvation with chloroquine. Gapdh and β-actin served as loading controls. Western blots of the HeLa WT and vtRNA1-1 knock-out cells shown here are identical to those from Fig. 3c and Fig. 4c of the main text and were put here again for a better comparison to the western blots of the M1 and M2 mutant cell lines.

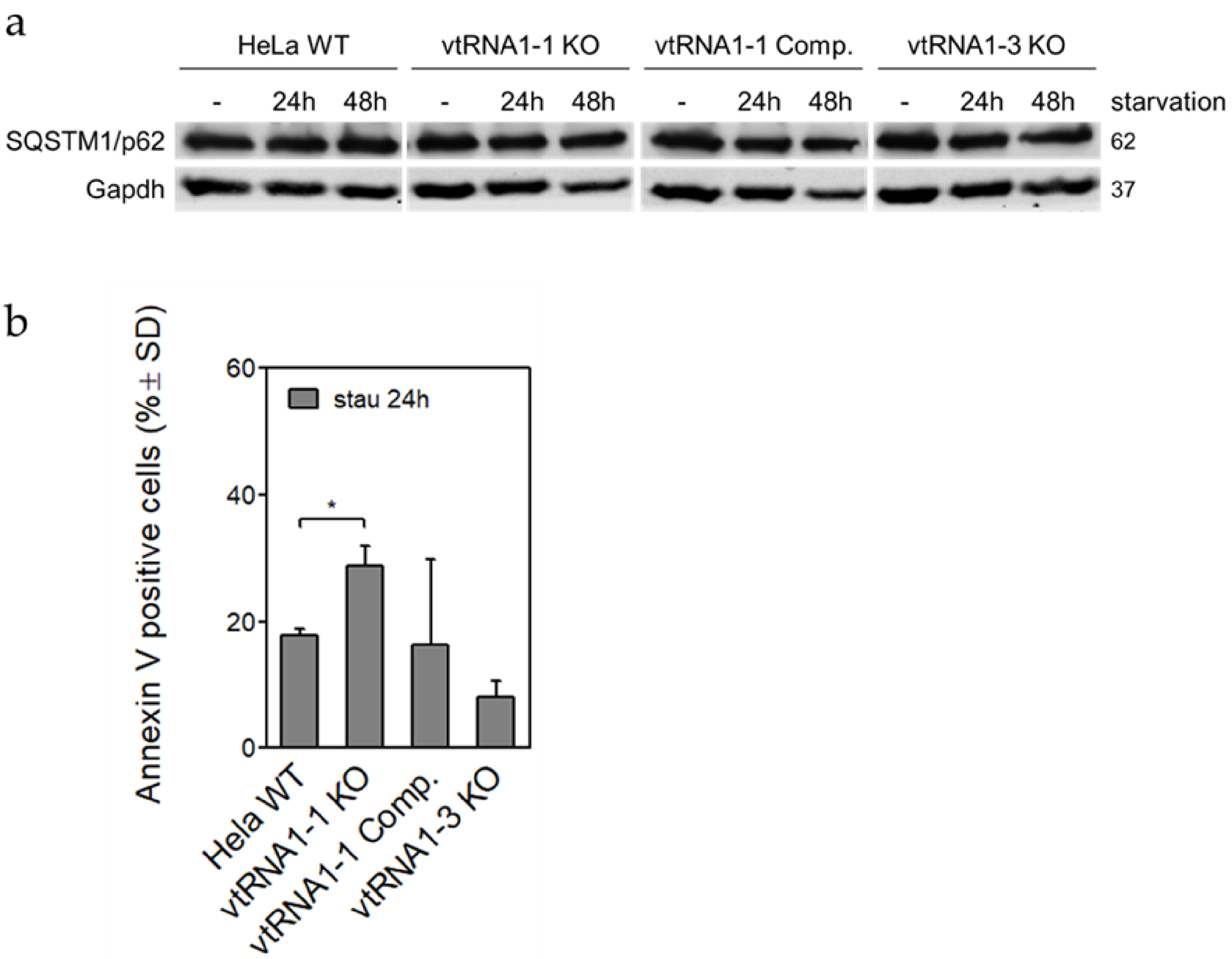

Supplementary Figure S6. (a) Western blot analysis of SQSTM1/p62 levels in HeLa WT, vtRNA1-1 knock-out, vtRNA1-1 complementation and vtRNA1-3 knock-out cells after 24 hours or 48 hours starvation in the presence of chloroquine. Gapdh served as loading control. **(b)** FACS quantification of annexin V positive HeLa WT, vtRNA1-1 knock-out, vtRNA1-1 complementation and vtRNA1-3 knock-out cells after 24 hours of staurosporine-induced intrinsic apoptosis. Significant differences were determined using the two-tailed unpaired Student's t-test (*P<0.01).

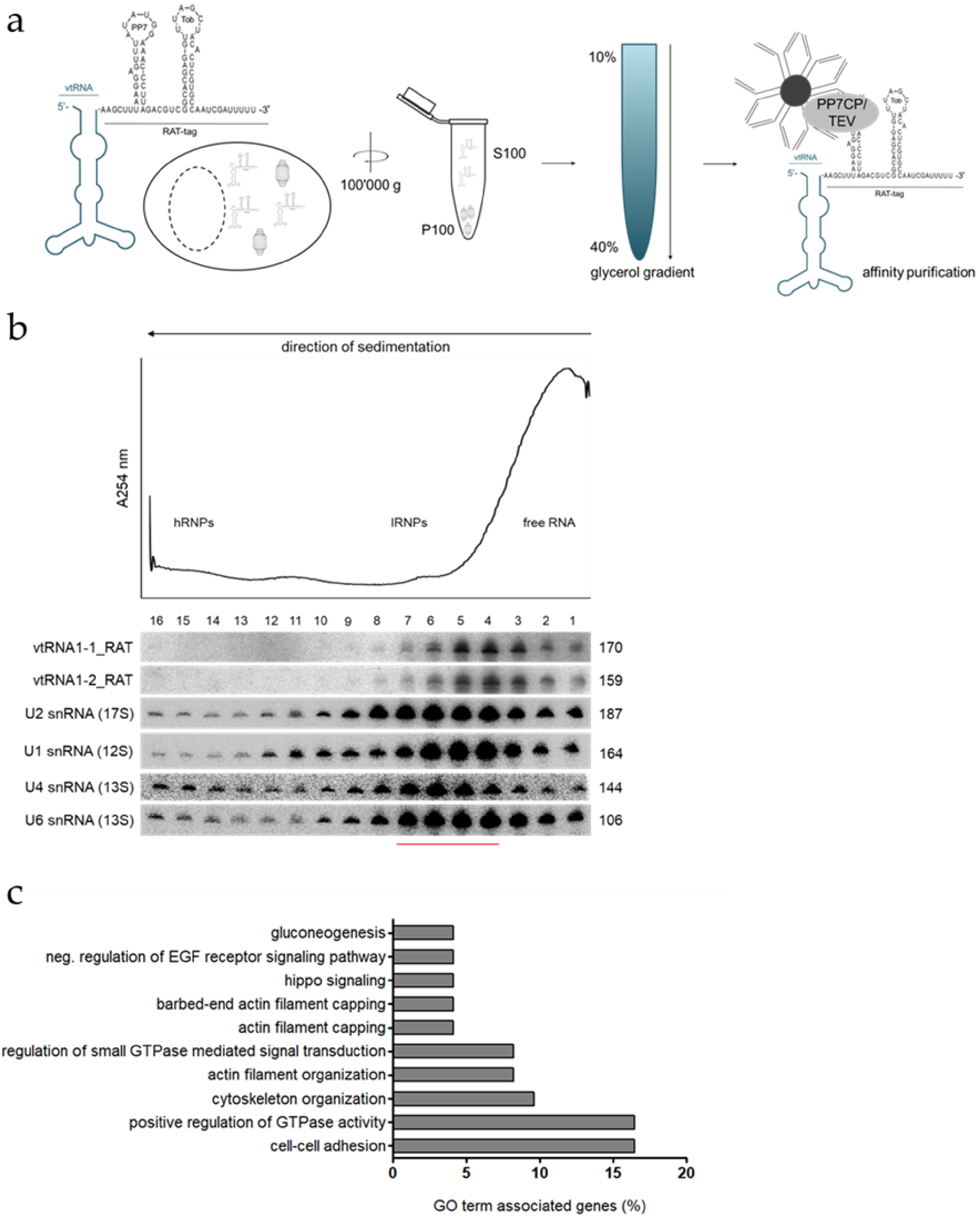

Supplementary Figure S7. Affinity purification of *in vivo* assembled vtRNA1-1-RNPs from BL41 cells **(a)** Schematic illustration of the experimental procedure. RAT-tagged vtRNA1-1, RAT-tagged vtRNA1-2 or the RAT-tag alone were stably expressed in BL41 cells. Cellular extracts were depleted by centrifugation at 100'000 xg from the large vault complex. The supernatant fraction (S100) containing smaller RNPs was separated on a 10% - 40% glycerol gradient. The affinity purification using the

recombinant *Pseudomonas aeruginosa* phage 7 coat protein (PP7CP) was performed on vtRNA positive fractions of the gradients. **(b)** Northern blot analysis of two control gradients in order to determine the localization of the RAT-tagged vtRNA1-1 and vtRNA1-2, respectively. The presence of spliceosomal U-rich small nuclear RNAs (snRNAs) was used to estimate the size and the sedimentation coefficient of the vtRNA-RNPs. vtRNA-positive fractions 4-7 were pooled and used for the RAT-tag affinity purification (red line). **(c)** Gene ontology (GO) analysis of LC-MS/MS identified vtRNA1-1 binding proteins. The top 73 candidates of vtRNA1-1 binding proteins from the LC-MS/MS analysis were selected according to their FDR value and subjected to the online database for annotation, visualization and integrated discovery (DAVID).
